# Cytogenetic Analysis of the Fish Genus *Carassius* Indicates Divergence, Fission, and Segmental Duplication as Drivers of Tandem Repeat and Microchromosome Evolution

**DOI:** 10.1093/gbe/evae028

**Published:** 2024-02-10

**Authors:** Nicola R Fornaini, Halina Černohorská, Lívia do Vale Martins, Martin Knytl

**Affiliations:** Department of Cell Biology, Faculty of Science, Charles University, Prague 12843, Czech Republic; Genetics and Reproductive Biotechnologies, CEITEC—Veterinary Research Institute, Brno 62100, Czech Republic; Department of Biology, Federal University of Piauí, Floriano, Piauí, Brazil; Department of Cell Biology, Faculty of Science, Charles University, Prague 12843, Czech Republic; Department of Biology, McMaster University, Hamilton, Ontario L8S4K1, Canada

**Keywords:** teleost fish, polyploidy, U1 and U2 snDNAs, histone H3, chromosome painting, FISH

## Abstract

Fishes of the genus *Carassius* are useful experimental vertebrate models for the study of evolutionary biology and cytogenetics. *Carassius* demonstrates diverse biological characteristics, such as variation in ploidy levels and chromosome numbers, and presence of microchromosomes. Those *Carassius* polyploids with ≥150 chromosomes have microchromosomes, but the origin of microchromosomes, especially in European populations, is unknown. We used cytogenetics to study evolution of tandem repeats (U1 and U2 small nuclear DNAs and H3 histone) and microchromosomes in *Carassius* from the Czech Republic. We tested the hypotheses whether the number of tandem repeats was affected by polyploidization or divergence between species and what mechanism drives evolution of microchromosomes. Tandem repeats were found in tetraploid and hexaploid *Carassius gibelio*, and tetraploid *Carassius auratus* and *Carassius carassius* in conserved numbers, with the exception of U1 small nuclear DNA in *C. auratus*. This conservation indicates reduction and/or loss in the number of copies per locus in hexaploids and may have occurred by divergence rather than polyploidization. To study the evolution of microchromosomes, we used the whole microchromosome painting probe from hexaploid *C. gibelio* and hybridized it to tetraploid and hexaploid *C. gibelio*, and tetraploid *C. auratus* and *C. carassius*. Our results revealed variation in the number of microchromosomes in hexaploids and indicated that the evolution of the *Carassius* karyotype is governed by macrochromosome fissions followed by segmental duplication in pericentromeric areas. These are potential mechanisms responsible for the presence of microchromosomes in *Carassius* hexaploids. Differential efficacy of one or both of these mechanisms in different tetraploids could ensure variability in chromosome number in polyploids in general.

**Significance**

Fish of the genus *Carassius* (Teleostei: Cyprinidae) are very popular models for studying genome and karyotype evolution, but there is a large gap in knowledge of microchromosome evolution and non-nucleolar tandemly repeated sequences at the level of experimental multispecies comparative studies. Based on cytogenetic investigation of closely related species with different ploidies, we propose three major processes that drive the evolution of microchromosomes and tandem arrays—divergence, fission, and post-polyploidization segmental duplication. All three drivers have a crucial impact on the evolution of *Carassius* and may trigger diversification into a plethora of individual extant species in the world.

## Introduction

In the fish family Cyprinidae (Cypriniformes, Teleostei), several independent polyploidization events (multiplications of haploid set of chromosomes) have occurred, giving rise to species with a large diversity of ploidy levels (Cherfas 1966). Ancestral number of chromosomes for the entire group of cyprinids (cyprinids, cyprinid group = the whole family Cyprinidae) is estimated to be 50 (Winfield and Nelson 2012).

Within Cyprinidae, the paleotetraploid clade Cyprinini (sensu Yang et al. 2010) includes the *Carassius* and *Cyprinus* genera, both of which have 100 chromosomes and solely *Carassius* has ∼150 and ∼200 chromosomes. It is clear that at least three polyploidization events occurred in this genus (since the independent evolution of the clade Cyprinini). The first polyploidization occurred in the common ancestor of Cyprinini and resulted in a chromosome number equal to 100 (Yang et al. 2010). The second polypolyploidization gave rise to the chromosome number ∼150 and the third to ∼200. Species from the clade Cyprinini that have 100 chromosomes are considered evolutionary tetraploids because of the most recent diploid ancestor did have 50 chromosomes (Ohno et al. 1967). In terms of biological development, these evolutionary tetraploids produce reduced gametes with 50 chromosomes that fuse during fertilization and the offspring continues to develop with recombinant and restored unreduced genetic information. The karyotype formula of a diploid member of Cyprinini shows biological and evolutionary ploidy by means of 2n=4x, where *n* defines the number of chromosomes in a gamete of the extant species (biological ploidy), and *x* refers to the number of chromosomes in a gamete of the most recent diploid ancestor of the extant species (evolutionary ploidy) (Knytl et al. 2017). In the following text, ploidy levels will be referred to the evolutionary term of ploidy, i.e. tetraploid, hexaploid, and octoploid *Carassius* are those with 100, ∼150, and ∼200 chromosomes, respectively.

*Carassius* is the most commonly used Cyprinini model for biological research (e.g. Pang et al. 2017; Ağdamar et al. 2020; Khosravi et al. 2022; Pavlov 2022a, 2022b; Tapkir et al. 2022; Wang et al. 2022a; Fedorčák et al. 2023; Jan et al. 2023; Tapkir et al. 2023). Its exceptional diversity of skills represents possible biological phenomena such as the presence of three ploidy levels—tetraploid, hexaploid, and octoploid (Kalous and Knytl 2011; Xiao et al. 2011; Knytl et al. 2022). The alternation of sexual and asexual (gynogenetic) mode of reproduction (Cherfas 1966; Przybył et al. 2020; Fuad et al. 2021) gives *Carassius* a competitive advantage in the rate of spatial expansion of asexuals. In addition, sexual reproduction should ensure higher resistance to parasites than gynogenesis due to recombination processes (Hakoyama et al. 2001). Another biological phenomena is sex determination, which in *Carassius* is governed by sex determining genes (Wen et al. 2020) and environmental temperature (Li et al. 2018). Variation in the number of chromosomes in *Carassius* polyploids (biologically speaking, individuals who possess ≥150 chromosomes) was revealed as another exceptional trait and may be caused by male genetic contribution into the egg/embryo (i.e. paternal leakage) during gynogenesis, leading to the presence of different numbers of microchromosomes in karyotypes (Yi et al. 2003; Ding et al. 2021). Macrochromosomes are larger than microchromosomes and possess clearly visible centromere, chromatids, and telomeres at both ends (Nanda and Schmid 1994).

Microchromosomes were originally thought to be redundant components of genomes, but have been found to be gene-rich and low in the content of the repetitive fraction (International Chicken Genome Sequencing Consortium 2004). In birds they occur in relatively high numbers (30–40 pairs) and their numbers are extremely conserved across various species (Waters et al. 2021; de Souza et al. 2023). However, this is not the case for *Carassius*. In *Carassius*, the number of microchromosomes ranges between 6 (Zhou and Gui 2002; Knytl et al. 2013b, 2018) and 18 (Zhao et al. 2021). There are only a few studies focusing on microchromosome painting, which usually crossed two strains of *Carassius* and analyzed their artificial offspring (Li et al. 2016, 2018; Zhao et al. 2021). The effect of paternal leakage in artificial *Carassius* progeny was evidenced by inseminating the *Carassius* egg with heterologous (i.e. from a species other than maternal one) sperm. The newly arisen offspring contained microchromosomes in karyotypes unlike the maternal karyotype, which did not contain them (Yi et al. 2003).

In the Czech Republic, *Carassius* is represented by four species. Commonly occurring invasive *Carassius gibelio* consists of tetraploid, hexaploid, and octoploid ploidy levels (Lusk et al. 2010; Knytl et al. 2013b). *Carassius auratus*, very well-known due to its colorful varieties as goldfish. This species also forms tetraploids, hexaploids, and octoploids (Xiao et al. 2011; Rylková et al. 2013). The third species, *Carassius carassius*, native to the Czech Republic, has been considered critically endangered since 2017 (Chobot and Němec 2017) and is strictly tetraploid (Knytl et al. 2013a). The fourth, *Carassius langsdorfii*, was discovered in the Czech Republic in 2007 by Kalous et al. (2007). The discovered female was hexaploid, but no cytogenetic examination other than conventional Giemsa staining has been conducted. Additionally, several *Carassius* hybrids were identified in natural Czech waters (Papoušek et al. 2008; Knytl et al. 2013b, 2018).

Due to the very complex characteristics and possible cryptic hybridization between *Carassius* species, it is difficult to reveal the origin of polyploids in the sense of allopolyploidy (more ancestors) or autopolyploidy (single ancestral species). Nevertheless, mixed allo- and autopolyploid origin was revealed and subgenomes were identified in hexaploid *C. gibelio* by whole genome sequencing (Kuhl et al. 2022). Subgenomes are genomic units that originate from lower ploidy ancestors. Genome of hexaploid *C. gibelio* consists of three subgenomes (Kuhl et al. 2022; Wang et al. 2022b). It is clear that hybridization and an allopolyploid event caused changes in the number of chromosomes and microchromosomes, but the origin and function of the microchromosomes in *Carassius* remain obscure, as well as the number and localization of the U1 and U2 loci of small nuclear DNA (snDNA) and/or histone H3, which represent the repetitive fraction of a genome by tandemly repeated arrays and are not associated with nucleolus (Huang and Spector 1992), hereafter referred to as (non-nucleolar) tandem repeats.

We used *Carassius* from natural waters of the Czech Republic to study inter-ploidy relationships within a species and between multiple species, specifically tetraploid and hexaploid *C. gibelio*, and tetraploid *C. auratus* and *C. carassius*. We used fluorescent in situ hybridization (FISH) to map U1 and U2 snDNAs and H3 histone in tetraploids and hexaploids. Moreover, we used painting FISH to map microchromosomes in hexaploids and genomic regions associated with microchromosomes in tetraploids. We addressed to answer the following questions: (i) Do interspecific *Carassius* tetraploids share the same number of non-nucleolar tandem repeats examined? (ii) Do hexaploid females have one and a half times higher proportion of these tandem repeat loci than tetraploids? (iii) Are there any differences in the number and position of FISH signals for microchromosomes in hexaploids? (iv) What are the possible origins of microchromosomes and the evolutionary forces behind their evolution? Here, we consider the distribution of tandem repeats and microchromosomes in *Carassius* tetraploid and hexaploid karyotypes in an evolutionary context.

## Results

### FISH with Repetitive DNA Probes

**Fig. 1.**
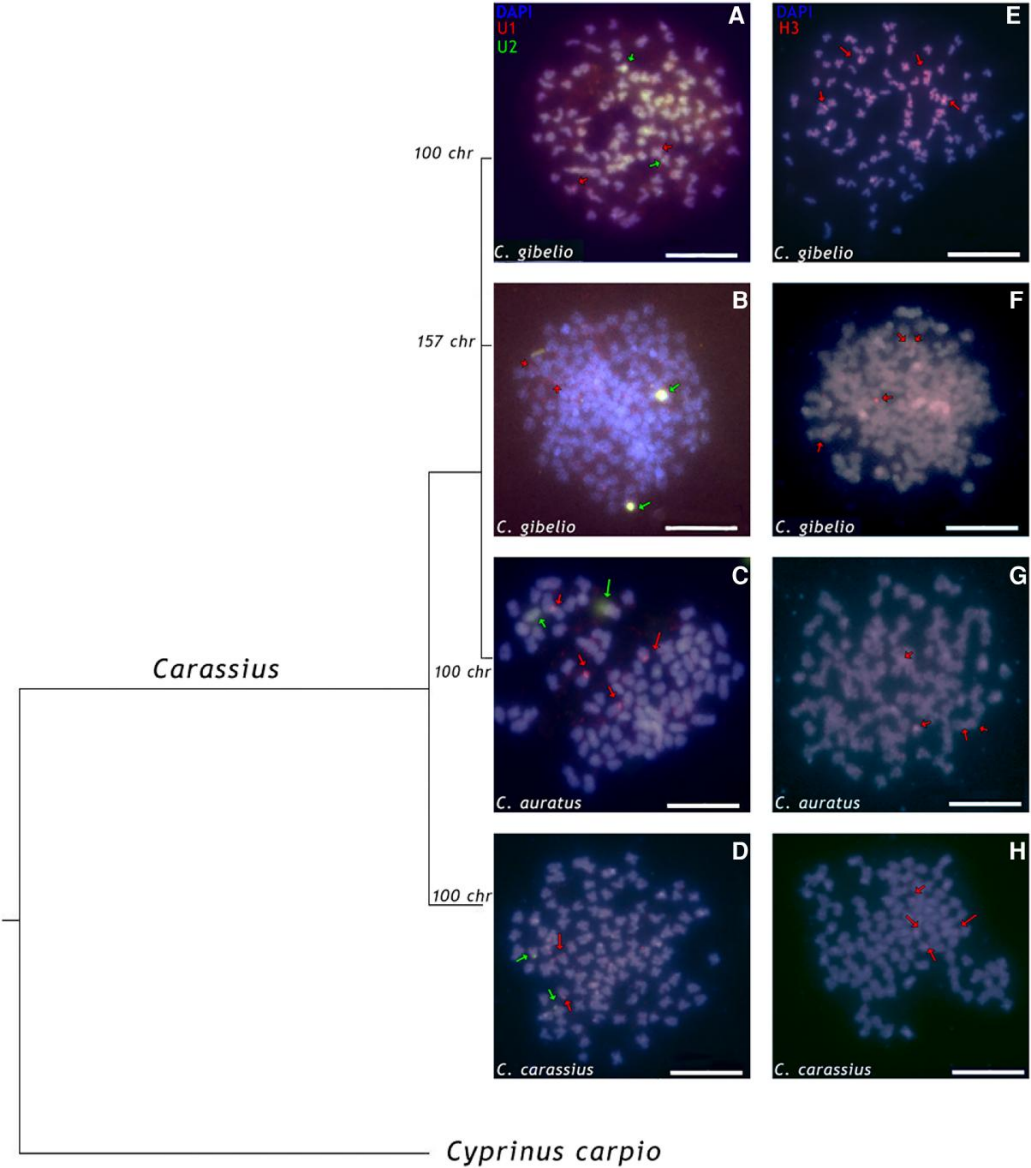
Double-color FISH with U1 and U2 snDNA probes (A, B, C, D). The U1 probe reveals one clear signal (= a pair of homologous chromosomes) in tetraploid (A) and hexaploid (B) *C. gibelio*, tetraploid *C. carassius* (D), while the same FISH shows two signals in tetraploid *C. auratus* (C). The U2 probe shows one signal in all species. The green and red arrows correspond to the U2 and U1 repeat loci, respectively. The U1 and U2 signals are located in the telomeric part of chromosomes as well as the H3 signals. Single-color FISH with the H3 snDNA probe (E, F, G, H). The probe shows two clear signals for all species: *C. gibelio* (E, F), *C. auratus* (G), and *C. carassius* (H). Chromosomes were counterstained with 4', 6-diamidino-2-phenylindole (DAPI) in blue/gray. Some DAPI-intensive spots are also visible at centromere positions or cover entire chromosomes (B, D, E, no arrows). Scale bars represent 10μm. Each *Carassius* metaphase is anchored in the phylogenetic tree to visualize the phylogenetic distance between specimens. Each branch depicts the number of chromosomes that corresponds to the appropriate individual. *Cyprinus carpio* is used as outgroup at the bottom of the figure.

We hybridized *C. gibelio* U1, U2, and H3 probes in four different groups: tetraploid (2n=4x=100) and hexaploid (3n=6n=157) *C. gibelio*, tetraploid *C. auratus* (2n=4x=100), and tetraploid *C. carassius* (2n=4x=100) (Fig. 1). Evolutionary relationships among the *Carassius* species examined are shown in the phylogenetic tree with *Cyprinus carpio* as an outgroup. *Carassius gibelio* and *C. auratus* form a single mitochondrial clade, while *C. carassius* is more distant to the others. FISH using U1 and U2 probes showed two signals (on one homologous pair) for each gene, except for the U1 gene in *C. auratus*, which showed four clear signals, while two signals for the U2 gene are consistent with the other species. FISH using H3 probe showed four signals in each species. The same number of loci did not support the expectation that hexaploid *C. gibelio* should have one and a half as many snDNA loci as tetraploid *C. gibelio*. These results indicate a copy number reduction or loss of the entire U1, U2, and H3 loci in a subgenome (after polyploidization). The variation in the number of FISH signals in individuals with the same ploidy level and the chromosome number may be explained by a variation in the copy numbers of tandem repeats per locus, which is explained in detail in section “snDNA Tandem Repeats”.

### Intra-ploidy Painting FISH with Whole Microchromosome Painting Probe

Microchromosomes selected for microdissection and subsequent FISH procedure were identified based on the following criteria: they are tiny, have an unclear centromere position and indistinguishable chromatids, and may be unpaired in a karyotype.

**Fig. 2.**
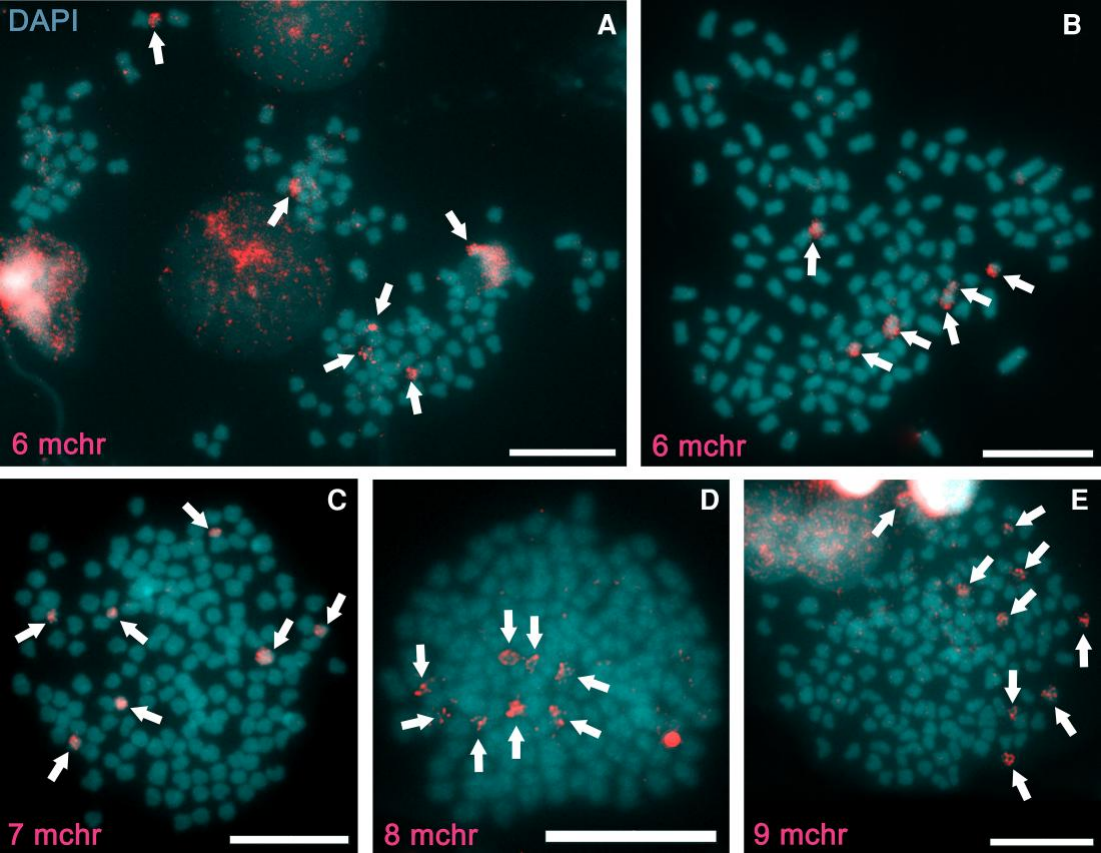
Intra-ploidy conspecific painting FISH with the *C. gibelio* whole microchromosome painting probe hybridized to the hexaploid *C. gibelio* females. Each metaphase represents a different individual. The microchromosome probe (red signal, arrows) shows different numbers of microchromosomes in each individual, respectively, six (A, B), seven (C), eight (D), and nine (E). Chromosomes were counterstained with DAPI in blue/gray. Nuclei with unfragmented chromatin are visible (top left, A, E). Scale bars represent 10μm.

The first painting FISH experiment was conspecific (*C. gibelio* probe against *C. gibelio* metaphases) and within individuals with the same ploidy level (hexaploid probe against hexaploid metaphases), referred to as intra-ploidy conspecific painting FISH. We conducted these experiments as a control to see if the whole microchromosome painting probe worked well, and also to see if the number of microchromosomes differed among *Carassius* populations. We used *Carassius* chromosomes from three different river basins in the Czech Republic. Conspecific painting FISH revealed variation in the number of microchromosomes, which ranged from six to nine (Fig. 2).

Five investigated females possessed 150 (Odra River basin, Fig. 2A), 149, 150 (both Elbe River basin, Fig. 2B,C), 150 (Mrlina River basin, Fig. 2D), and 153 (Elbe River basin, Fig. 2E) chromosomes with 6, 6, 7, 8, and 9 microchromosomes, respectively. *Carassius* used to generate the whole chromosome painting probe originated from the Odra River basin. The variation in the number of microchromosomes was consistent with previous studies (Zhou and Gui 2002; Knytl et al. 2013b; Li et al. 2016; Knytl et al. 2018; Li et al. 2018). The FISH signal was consistently spread over the entire surface of the microchromosomes, indicating the high efficacy of the FISH technique conducted. The presence of clearly highlighted chromosomes indicates that the probe and competitor DNA collaborated properly and that nonspecific hybridization of the probe to repetitive regions was inhibited. The *Carassius* microchromosomes did not show a high degree of heterochromatization, as is typical for B chromosomes, which are sometimes considered to be microchromosomes (Bishani et al. 2021; Gvoždík et al. 2023). Our finding is consistent with a high gene content and a low proportion of a repetitive fraction within the microchromosomes.

### Inter-ploidy Painting FISH with Whole Microchromosome Painting Probe

**Fig. 3.**
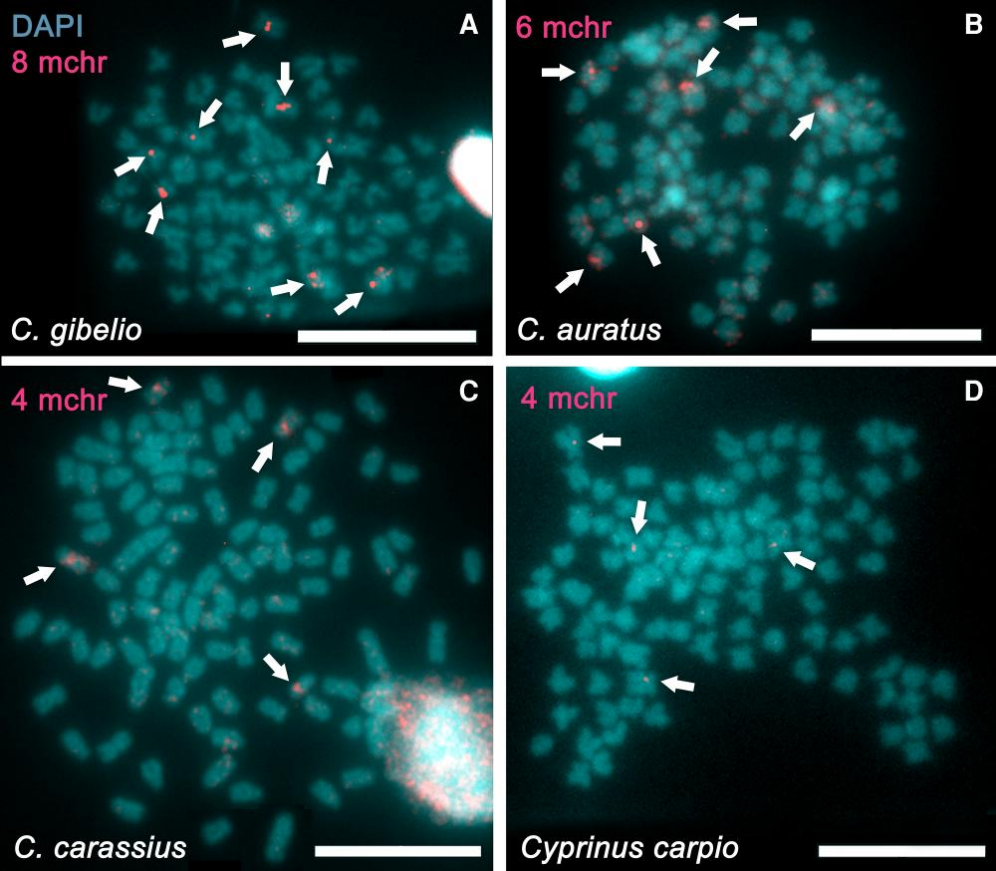
Inter-ploidy painting FISH with the *C. gibelio* whole microchromosome painting probe mapped to chromosomes of four tetraploid species, *C. gibelio*, *C. auratus*, *C. carassius*, and *C. carpio*. The microchromosome probe (red) shows signals on portions of the pericentromeric chromosomal regions, indicated by arrows. The number of FISH signals varies by species: eight in *C. gibelio* (A), six in *C. auratus* (B), four in *C. carassius* (C), and four in *C. carpio* (D). Chromosomes were counterstained with DAPI in blue/gray. Scale bars represent 10μm.

The other aim of the study was to trace the origin of microchromosomes using tetraploid *Carassius* relatives and to determine whether some of the genome sequences in tetraploids are similar to those sequences in microchromosomes in hexaploids. We hybridized the whole microchromosome painting probe (the same one as we used for intra-ploidy painting FISH) to metaphase spreads of tetraploid male and female *C. gibelio* (inter-ploidy conspecific painting FISH), *C. auratus*, *C. carassius*, and *C. carpio* (inter-ploidy interspecific painting FISH). Inter-ploidy painting FISH showed the similarity of microchromosomes to some genomic regions in tetraploids (microchromosome-associated regions). Fluorescent signals do not cover the entire chromosome lengths and are usually located in pericentromeric regions of mapped chromosomes. The highlighted pericentromeric region indicates that this portion gave rise to the microchromosomes by fission of macrochromosomes, followed by segmental duplication of specific pericentromeric regions (see in “Mechanism of Origin of Carassius Microchromosomes”). The number and intensity of the FISH signals decrease with increasing phylogenetic distance (Fig. 3). As expected, *C. gibelio* (Fig. 3A) showed the highest intensity and the highest number of the FISH signals. On the other hand, *C. carpio* (Fig. 3D), the most distant relative, showed very low intensity of the FISH signals. The number of signals ranges from 8 to 10 in *C. gibelio*, from 6 to 8 in *C. auratus*, and from 4 to 6 in *C. carassius*. The number of signals in *C. carpio* was stable and showed four loci related to the microchromosome-associated regions. No differences between males and females were found.

Considering the mapping of tandem repeats and microchromosomes together, we can exclude co-localization of the investigated tandem repeats with microchromosome-associated regions, and thus we can exclude mutual interactions of U1 or U2 snDNAs or H3 histones with microchromosome-associated regions within a single chromosome. The FISH signals of tandem repeats are situated in pericentromeric regions of telocentric and/or subtelocentric chromosomes with measured centromeric index 0–25. Microchromosome-associated fragments are also located in pericentromeric regions but on submetacentric and metacentric chromosomes (centromeric index 25–50). In each *Carassius* species, a pair of chromosomes carrying the microchromosome-associated locus is one of the largest chromosomes in the karyotype.

## Discussion

### snDNA Tandem Repeats

*Carassius* is a widely used experimental model for its extraordinary characteristics, but is rarely used for tandem repeat mapping or microchromosome painting. The consequences of genome duplication and divergence can be studied if we can compare the number of tandem repeat loci between different ploidy levels of closely related species (Fornaini et al. 2023), and this is precisely what can be examined in *Carassius*.

One way to find out these evolutionary consequences is to map tandem repeats on chromosomes using FISH. We compared the number of FISH signals of non-nucleolar tandem repeats (U1 and U2 snDNA and H3 histone) in tetraploid *C. gibelio* with hexaploid *C. gibelio*. Our hypothesis was that hexaploid *C. gibelio* would have one and a half as many signals as tetraploid *C. gibelio*. However, we found equal numbers of all tandem repeat loci in both tetraploids and hexaploids (Fig. 1). The expectations were not met, and we found out that divergence affected the number of tandem repeats more effectively than polyploidization. Since we found the same number of tandem repeats in *C. gibelio* tetraploids and hexaploids, hexaploids may experience a reduction in the number of tandem repeat copies per locus or a complete loss of a tandem repeat locus, likely due to an ongoing evolutionary process of re-diploidization with no need to duplicate and produce more snRNA or H3 histones than in tetraploids (Fornaini et al. 2023). In the other investigated tetraploids, *C. auratus* and *C. carassius*, we found the same number of tandem repeats as in hexaploid *C. gibelio*, except for the U1 locus in *C. auratus* localized on two chromosome pairs. The number of mapped loci is summarized in Table 1.

**Table 1:** Numbers of U1 and U2 SnDNA, histone H3, and microchromosome-associated (Mchr.) loci in *Carassius* found in this study

Specimen	Ploidy	U1	U2	H3	Mchr.
*C. gibelio*	2n=4x	2	2	4	8–10
*C. gibelio*	3n=6x	2	2	4	6–9
*C. auratus*	2n=4x	4	2	4	6–8
*C. carassius*	2n=4x	2	2	4	4–6
*C. carpio*	2n=4x	NA	NA	NA	4

*Note*. Signals are counted per whole genome (one locus = signal on one chromosome regardless of homologous or non-homologous). NA = information not available from this study.

Mapping of snDNAs is rare throughout Cyprinidae. To our knowledge, this is the first cytogenetic localization of U1 snDNA within the clade Cyprinini. For instance, in the cyprinid genus *Hypophthalmichthys*, which is not paleotetraploid as Cyprinini, a single chromosome pair carries the U1 snDNA locus (Sember et al. 2020). Why the U1 locus was retained duplicated in *C. auratus* and why this locus was reduced in other *Carassius* species is not yet clear, but few other known U1 mapping studies carried out on species other than cyprinids show that the U1 locus is present in a karyotype on single chromosome pair (Cabral-De-Mello et al. 2012; Carvalho et al. 2017; Malimpensa et al. 2020) as well as on six chromosome pairs (Silva et al. 2015). The U2 snDNA locus was previously mapped to *C. carassius* and *C. gibelio* (Bishani et al. 2021). The number of the U2 snDNA signals in *C. carassius* agrees with our results, however, Bishani et al. (2021) identified six U2 signals (two triplets) in the hexaploid *C. gibelio* versus two signals we found in this study. This inconsistency may be due to genetic variation in the copy number of U2 tandem repeats per locus between different *C. gibelio* populations because *C. gibelio* is a complex of individuals with diverse biological skills and different origins (Knytl et al. 2022; Lu et al. 2023).

Another type of tandem repeats is ribosomal RNA which underpins and organizes the nucleolar organizer structure (NOR) and contains 18S, 5.8S, and 28S clusters of ribosomal DNA, rDNA (nucleolar tandem repeats) (Symonová and Howell 2018). The number of nucleolar tandem repeats in tetraploid *Carassius* appears to be more complex. Tetraploid *Carassius* has NORs on two chromosome pairs (Spoz et al. 2014; Knytl et al. 2018; Knytl and Fornaini 2021) which indicates the multiplication of the NOR number due to whole genome duplication and is consistent with paleotetraploid origin (Yang et al. 2010). Cytogenetic mapping of nucleolar tandem repeats in *Carassius* hexaploids indicates a complete/incomplete loss of this locus in one subgenome because their number in hexaploid is equal to the number of NORs in tetraploids (Knytl et al. 2018).

Both nucleolar and non-nucleolar tandem repeats have similar evolutionary destinies leading to loss and/or deletion of redundant copies or reduced copy number per locus—one pair of U1, one pair of U2 snDNAs, and two pairs of H3 histones in tetraploid and hexaploid *C. gibelio* (this study); two pairs of NORs in tetraploid and hexaploid *Carassius* (Knytl et al. 2018).

### Evolution of *Carassius* Microchromosomes

The complex of the genus *Carassius* is a group of representatives with different characteristics and in some cases they do not meet the definition of a species (Kalous et al. 2012; Knytl et al. 2022). Therefore, it is important to comprehend the phylogeny of this group. For this reason, we generated a phylogenetic tree from the known mitochondrial sequences of the cytochrome b gene. This analysis confirmed the closest relationship between *C. gibelio* and *C. auratus* and a greater evolutionary distance between the latter two species and *C. carassius* (phylogenetic tree in Fig. 1). Chromosome painting with the whole microchromosome painting probe coincides with the phylogenetic distance and shows the highest intensity and number of signals in the most closely related tetraploid *C. gibelio* and *C. auratus* (Fig. 3A, B and Table 1). Tetraploid and hexaploid *C. gibelio* can form polyphyletic lineage (Kalous et al. 2012), and since there is a diversity of abilities between these ploidy levels, such as origin and modes of reproduction, definition of species is somewhat of controversial (Kalous et al. 2012). When we used the whole microchromosome painting probe on *C. carassius* chromosomes, the intensity and number of fluorescent signals are obviously lower than on *C. gibelio* and *C. auratus* chromosomes, but spatially the signal in *C. carassius* covers at least the same-size or even larger chromosomal region than in *C. gibelio*/*C. auratus* (Fig. 3A–C). In the most distant *C. carpio*, intensity of the signal is the lowest from all specimens tested, and the number of signals is equal to the number of signals in *C. carassius*.

High intensity and strength of the FISH signal may be indicative of repetitive elements, as is the case of tandem repeats in this study or a different type of repetitions in other studies (e.g. Knytl et al. 2018). We tried to avoid a repetitive signal by using autoclaved competitor DNA. This DNA blocking approach inhibits binding of a probe to repetitions. We successfully used the same DNA blocking technique for painting FISH to map unique genomic sequences (Knytl et al. 2013b, 2017, 2023). We also increased stringency washing because signals were not detected using the previously verified FISH protocol. Using the described DNA blocking and protocol modifications, we exclude random nonspecific binding of the probe to chromosomes.

It has been proposed that the number of microchromosomes varies within *Carassius* species (Yi et al. 2003; Li et al. 2016, 2018) and microchromosomes are one of the sources associated with variation in chromosome number in *Carassius*. Yi et al. (2003) carried out a crossbreeding experiment in which *C. gibelio* eggs were fertilized with heterologous sperm of *Megalobrama amblycephala*. The newly produced artificial progeny possessed from 5 to 15 microchromosomes. The microchromosomes were microdissected and used for whole microchromosome painting probe. The probe was hybridized with chromosomes of both parental species. The maternal karyotype did not show any FISH signals, indicating absence of microchromosomes. The paternal karyotype of *M. amblycephala* contained microchromosome-associated regions on four chromosome pairs and the signal was spread over parts of the chromosomes. Two signals were situated on telomeres and the remaining two signals in pericentromeric regions. The study brought evidence of paternal leakage into the allogynogenetic offspring. Similarly, we observed signals in the pericentromeric regions of two to four pairs of chromosomes in *Carassius* tetraploids and *C. carpio*.

### Mechanism of Origin of *Carassius* Microchromosomes

If the microchromosomes in the hexaploid females we examined were derived from the paternal leakage, all six to nine microchromosomes would have originated from the father, as suggested by the artificial crosses conducted by Yi et al. (2003). There is no evidence as to what species was the donor of the microchromosomal genome complement in our study. Both maternal genome complement and paternal leakage could have led to the synergistic collaboration and microchromosome formation in hexaploids. The number of microchromosomes can shape/change independently multiple times due to paternal leakage through gynogenesis (Yi et al. 2003; Lamatsch and Stöck 2009; Knytl et al. 2013b). It was found that microchromosomes are building blocks in birds, reptiles, and mammals that undergo the mechanism of macrochromosome fission and/or fusion of two micro- or micro- and macrochromosomes (Kretschmer et al. 2020; Waters et al. 2021). For instance, mammals do not have microchromosomes (Srikulnath et al. 2021) and therefore fusions involving microchromosomes occurred in their common progenitor(s), but after divergence with birds and reptiles that do have microchromosomes (Waters et al. 2021). In *Carassius*, the most likely mechanism for the origin of microchromosome is fission of macrochromosomes. Our results indicate that microchromosomes arose by fission from the pericentromeric regions of the submeta- and metacentric chromosomes of tetraploid *Carassius* species.

The number of microchromosomes in hexaploid females (6–9) corresponds to the number of microchromosome-associated regions in tetraploids (4–10), but the painting signal in tetraploids covers only a portion of the chromosomal area compared to the signal on entire chromosomes in hexaploids, so there should be an additional mechanism besides fission that amplifies microchromosome-like regions. Duplication of an entire block of gene(s) translocated from the macrochromosome to the microchromosome after genome duplication, i.e. segmental duplication (Leister 2004), might be an accompanying mechanism that played an important role in the evolution of *Carassius* microchromosomes. Presumably, tandem duplication, a process in which gene(s) is/are replicated at the original chromosomal locus without subsequent relocation (Blanc et al. 2000), might collaborate with fission after whole genome duplication in *Carassius*. Segmental or tandem duplications can be followed by gene loss at the original locus (Leister 2004) due to divergence (see less widespread FISH signals in tetraploids than in hexaploids; Figs. 2 and 3). Segmental/tandem duplications were evidenced, for example, in plants of the genus *Arabidopsis* (Blanc et al. 2000; Cannon et al. 2004; Leister 2004).

Direct proof of our proposed mechanisms can be provided by sequencing of *Carassius* microchromosomes. This analysis requires microdissection, library preparation, and sequencing of every single microchromosome. While this analysis is time consuming and costly, future evidence is desirable for a better mechanistic understanding of the evolution of *Carassius* microchromosomes. Since *Carassius* genomes have already been sequenced (Li et al. 2021; Kuhl et al. 2022; Wang et al. 2022b), it is possible to map sequences from microdissected microchromosomes and compare them with microchromosome sequences from Chinese *Carassius* populations (Ding et al. 2021).

### Back in Time: Microchromosomes in the Most Recent Common Ancestor of Bony Vertebrates

Bony vertebrates (Euteleostomi, sensu lato Osteichthyes) are a clade of vertebrates that formed after divergence from agnathans and chondrichthyians. It is estimated that the ancestral karyotype of bony vertebrates is similar to that of spotted gar (*Lepisosteus oculatus*). The present-day karyotype of this species contains 58 chromosomes, involving macrochromosomes and from 18 to 20 microchromosomes (Braasch et al. 2016; Symonová et al. 2017). *Lepisosteus oculatus* represents a strong conservation of microchromosome structure and synteny over 450 million years, for example, compared to the chicken genome (Braasch et al. 2016). It is assumed that the ancestral karyotype of bony vertebrates contained 12 microchromosomes (Sacerdot et al. 2018), a number similar to that of *Carassius*. It is questionable whether *Carassius* microchromosomes arose independently from macrochromosomes of *Carassius* tetraploids, or whether these microchromosomes are highly conserved and homologous to the microchromosomes of ancestor of bony vertebrates, eventually to those of chicken and gar. Our results suggest an independent origin of the *Carassius* microchromosomes, as neither *Carassius* tetraploids nor *C. carpio* have any microchromosomes, but this does not exclude the hypothesis that *Carassius* microchromosomes are homologous to microchromosomes of chicken and/or gar. Microchromosomes can arise and disappear spontaneously during evolution due to the fusion–fission model of macro- and microchromosomes (reviewed in Srikulnath et al. 2021). Mapping *Carassius* microchromosomes to genomes that are not only closely related to *Carassius* genomes, but also to genomes related to the most recent common ancestor of bony vertebrates, i.e. the basal lineages of gars, is a challenge for further *Carassius* research.

## Conclusion

Both repetitive elements and microchromosomes are integral components of the *Carassius* genome and their evolution can be affected by polyploidization or divergence between species. Analysis of tandem repeat mapping showed evolutionary conservation in the number of these mapped repeats between *Carassius* species with different ploidy levels, except for the U1 snDNA locus in *C. auratus*. This conservation suggests a reduction and loss of copy number per locus (particularly in hexaploids) that may have occurred by divergence after polyploidization. For further examples of reduction, loss, and/or expansion of copy number of tandem repeats per locus, see, e.g. Fornaini et al. (2023). Analysis of the whole microchromosome painting revealed evolution by fission followed by post-polyploidization segmental duplication of pericentromeric macrochromosomal regions as a potential mechanism responsible for the presence of microchromosomes in *Carassius* hexaploids. Other examples of these latter evolutionary mechanisms are Cannon et al. (2004) and Kretschmer et al. (2020). We did not find co-localization of tandem repeats with microchromosome-associated regions, so we ruled out their cooperation within the same chromosome locus. Both structures evolved independently in *Carassius*. All of our findings are consistent with structural genomic changes that follow genome duplication.

## Materials and Methods

### Fish Sampling and Origin

*Carassius auratus* was obtained via the aquarium trade (transported to the Czech Republic from Israel). *Carassius carassius* was collected in alluvial ponds and old oxbows of the Elbe River basin close to the city Lysá nad Labem, the Bohemia region. *Carassius gibelio* was captured in the Elbe River basin close to the city Lysá nad Labem, the Bohemia region (hexaploids); in the Mrlina River basin, Global Positioning System: 50 ^∘^15 ^′^35.8 ^″^N, 15 ^∘^08 ^′^43.4 ^″^E, the village Zábrdovice, the Bohemia region (hexaploids); in the Odra River basin, the Moravian-Silesian region (tetraploids, hexaploids). All three river basin districts are located in the Czech Republic. Field surveys of ichthyofauna were performed in 2010–2012 and then in the River Mrlina in 2021. More detailed information on morphological analysis, voucher specimens, and location coordinates are given in Knytl et al. (2013b). Sex of individuals was identified based on dissection of gonads. Males and females of *Carassius* tetraploids and females of *Carassius* hexaploids were used in this study. *Cyprinus carpio* gonads were not inspected by dissection and sex of this individual was not identified. Because *C. carpio* was used as an outgroup for our analyses, knowledge of sex and origin is not crucial to our investigation.

### Chromosome Preparations

Chromosome spreads were prepared from tetraploid males and females (*C. gibelio*, *C. auratus*, and *C. carassius*) and hexaploid *C. gibelio* females. For all *Carassius* species, we used a method in which chromosome suspension was prepared directly from the cephalic kidney (Bertollo and Cioffi 2015). Mitotic activity was stimulated by intraperitoneal injection of 0.1% CoCl _2_ per 100 g of weight 24 h before colchicine (Sigma, St. Louis, MO, USA) application (Knytl et al. 2018). Ready-to-use chromosome suspension was stored in fixative solution (methanol:acetic acid, 3:1, v/v) at −20 °C as described in Knytl and Fornaini (2021). *Cyprinus carpio* chromosomes were obtained from regenerating fin tissues according to Kalous et al. (2010). The methods of colchicine treatment and hypotonization were originally adopted from *Chromaphyosemion* killifishes (Cyprinodontiformes, Nothobranchiidae) (Völker et al. 2006). Tissue fixation was performed and metaphases were spread on slides in the same manner as described in the embryo preparation protocol in Völker and Kullmann (2006). Microscopy and processing of metaphase images were conducted using Leica Microsystem (Wetzlar, Germany) as detailed in Seroussi et al. (2019). At least 20 metaphases were analyzed per each individual and five individuals were investigated per each analysis.

### FISH with Repetitive DNA Probes

In order to generate probes for FISH, genomic DNA (gDNA) from hexaploid *C. gibelio* was used as a template for amplification of the U1 and U2 snDNA regions, and H3 histone. DNA was extracted from adult fish tissues using the DNeasy Blood and Tissue Kit (Qiagen, Hilden, Germany) according to the manufacturer’s instructions. Primers used for amplification are listed in Table 2. The annealing temperature was 54 °C and the elongation step 30 s for all polymerase chain reactions (PCR); other conditions for PCR amplification with PPP Master Mix (Top-Bio, Prague, Czech Republic) followed the manufacturer’s recommendations. PCR amplification of the U1, U2, and H3 genes consistently resulted in 112, 140, and 375 bp long fragments, respectively. The search using the blastn algorithm confirmed the locus- and species-specificity of each amplicon: 98.98% identity with the U1 DNA sequence of *C. gibelio* (accession number XR_008182931.1), 97.10% identity with the U2 DNA sequence of *C. gibelio* (accession number XR_008154662.1), and 97.53% identity with H3 DNA of *C. gibelio* (accession number XM_052561945.1). Labeling PCR was performed as described in Knytl and Fornaini (2021). Digoxigenin-11-deoxyuridine triphosphate (dUTP) (Jena Bioscience, Jena, Germany) was used for U2 and H3 labeling, and biotin-16-dUTP (Jena Bioscience) was used for U1 labeling. *Carassius gibelio* U1 and U2 snDNA and H3 probes were then hybridized to chromosome spreads of *C. carassius* and *C. auratus* and tetraploid and hexaploid *C. gibelio*. The procedures for hybridization mixture preparation, denaturation, and subsequent overnight hybridization were previously described for rDNA FISH (Knytl et al. 2023). Post-hybridization stringency washing and blocking reactions were performed as described for painting FISH in Krylov et al. (2010). Probe signal was visualized following Knytl et al. (2017).

**Table 2:** Tandem repeats used for FISH analysis, their GenBank accession numbers, lengths, sequences of primers, and studies in which primers were designed

Gene name	Accession no.	Length (bp)	Primer sequence	Reference
Small nuclear RNA U1	OR366029	112	U1F 5^′^--GCAGTCGAGATTCCCACATT--3^′^	Silva et al. (2015)
			U1R 5^′^--CTTACCTGGCAGGGGAGATA--3^′^	
Small nuclear RNA U2	OR366030	140	U2F 5^′^--ATCGCTTCTCGGCCTTAT--3^′^	Bueno et al. (2013)
			U2R 5^′^--TCCCGGCGGTACTGCAATA--3^′^	
Histone H3	OR596332	375	H3F 5^′^--ATGGCTCGTACCAAGCAGAC(ACG)GC--3^′^	Colgan et al. (1998)
			H3R 5^′^--ATATCCTT(AG)GGCAT(AG)AT(AG)GTGAC--3^′^	

### Phylogenetic Tree

We used mitochondrial cytochrome b of *C. carassius* (accession number KR131839), *C. auratus* (accession number KX688781), diploid (tetraploid) *C. gibelio* (accession number KX688784), and triploid (hexaploid) *C. gibelio* (accession number KX601125.1) to construct the phylogenetic tree. The *C. carpio* mitochondrial genome (accession number NC_001606) was used as an outgroup. Sequences were aligned using the multiple sequence comparison by log-expectation (MUSCLE) algorithm (Edgar 2004) through MEGA11 software (Tamura et al. 2021). After alignment, Iq-tree2 software (Minh et al. 2020) was used to predict the nucleotide substitution model using ModelFinder (Kalyaanamoorthy et al. 2017) and to create the tree. The best fitting model was found to be Hasegawa-Kishino-Yano (HKY+F) (Hasegawa et al. 1985). The tree was displayed using FigTree http://tree.bio.ed.ac.uk/software/figtree/.

### Painting FISH with Whole Microchromosome Painting Probe

Microchromosomes from a single *C. gibelio* female were isolated individually by laser microdissection as previously described in Kubickova et al. (2002) using a PALM Microlaser system (Carl Zeiss MicroImaging GmbH, Munich, Germany). A total of 10 single microchromosomes were dissected from multiple metaphases (approximately two microchromosomes from each metaphase). These microchromosomes were then pooled and used to paint a whole microchromosome FISH probe, subsequently completed using the GenomePlex Single Cell whole genome amplification Kit (WGA4), Sigma-Aldrich, according to the manufacturer’s protocol for whole genome amplification with extracted gDNA. GenomePlex WGA Reamplification (WGA3), Sigma-Aldrich, and labeling with digoxigenin-11-dUTP (Jena Bioscience) were carried out as described in Krylov et al. (2010). Autoclaved *C. gibelio* gDNA (Bi and Bogart 2006) was used as a competitor (blocking DNA). The digoxigenin-labeled probe was detected by anti-digoxigenin-fluorescein (Roche, Basel, Switzerland). Conspecific painting FISH *C. gibelio*–*C. gibelio* was conducted as detailed in painting FISH in Krylov et al. (2010). Inter-ploidy painting FISH was carried out as described in Zoo-FISH in Krylov et al. (2010), with minor changes (Knytl et al. 2017). In addition, the current protocol was modified by increasing the total salt concentration from 2xSCC to 4xSCC (8xSCC with 50% formamide, 1:1, v/v) for less effective stringency washing. Chromosomes were counterstained with ProLong^TM^ Diamond Antifade Mountant with the fluorescent 4^′^,6-diamidino-2-phenylindole (DAPI) stain (Invitrogen by Thermo Fisher Scientific, Waltham, MA, USA).

## Data Availability

All the data supporting the findings of this study are available within the article. Sanger sequencing data are deposited in the GenBank NCBI database, https://www.ncbi.nlm.nih.gov/genbank/ (see Table 2 for accession numbers).
